# Usefulness of three‐dimensional thoracoscope for prone position thoracoscopic esophagectomy improves mediastinal lymph node dissection and prognosis for esophageal cancer

**DOI:** 10.1002/cnr2.1850

**Published:** 2023-06-20

**Authors:** Kohei Kanamori, Kazuo Koyanagi, Soji Ozawa, Junya Oguma, Akihito Kazuno, Yamato Ninomiya, Miho Yamamoto, Yoshiaki Shoji, Kentaro Yatabe, Masaki Mori

**Affiliations:** ^1^ Department of Gastroenterological Surgery Tokai University School of Medicine Isehara Japan; ^2^ Department of Esophageal Surgery National Cancer Center Hospital Tokyo Japan

**Keywords:** esophagectomy, lymph node dissection, prognosis, prone position, three‐dimensional thoracoscope

## Abstract

**Objectives:**

This study aimed to assess the superiority of 3D flexible thoracoscope against 2D thoracoscope for lymph node dissection (LND) and prognosis for prone‐position thoracoscopic esophagectomy (TE) in esophageal cancer.

**Methods:**

Three hundred and sixty‐seven esophageal cancer patients who underwent prone‐position TE with 3‐field LND between 2009 and 2018 were evaluated. 2D and 3D thoracoscope was used in 182 (2D group) and 185 cases (3D group), respectively. Short‐term surgical outcomes, numbers of retrieved mediastinal lymph node (LN), and rates of LN recurrence were compared. Risk factors for mediastinal LN recurrence and long‐time prognosis were also evaluated.

**Results:**

No differences in postoperative complications were observed between the groups. The numbers of retrieved mediastinal LN were significantly higher, and the rates of LN recurrence were significantly lower in the 3D group compared to 2D group. Use of 2D thoracoscope was a significant independent factor of middle mediastinal LN recurrence by multivariable analysis. Survival was compared by cox regression analysis, and the 3D group had a significantly better prognosis than the 2D group.

**Conclusions:**

Prone position TE using 3D thoracoscope may improve the accuracy of mediastinal LND and prognosis without increasing postoperative complications for esophageal cancer.

## 1 INTRODUCTION

Multimodal treatment with a combination of surgery, radiation, and chemotherapy is essential for improving prognosis for esophageal cancer patients.^1^ Among them, surgery is the mainstay of curative treatment, especially for resectable esophageal cancer.^2^ Esophagectomy with three‐field lymph node dissection (LND) has been proven to improve esophageal patients' prognosis^3^, ^4^, ^5^, ^6^ and therefore widely applied in Japan. The most frequent metastasis in thoracic esophageal cancer is mediastinal LN, therefore, an adequate dissection of mediastinal LNs is expected to improve outcomes.^7^, ^8^

Development of endoscopic surgery has shifted the esophagectomy procedures from open thoracotomy to thoracoscopy approach.^9^, ^10^ The magnification provided by the thoracoscope increased the accuracy of LND.^11^ Three‐dimensional (3D) endoscope had been developed recently to further improve surgical procedures.^12^ In Japan, the clinical use of 3D endoscope began around 2013, and then have been widely introduced for thoracoscopic esophagectomy (TE). The stereoscopic visibility and depth perception of the 3D endoscope are considered suitable for mediastinal LND for esophageal cancer. Although several studies had reported the utility of 3D endoscope in treating esophageal cancer,^13^, ^14^, ^15^ the clinical impact of 3D endoscope on LND accuracy, prevention of LN recurrence, and the contribution to patients' prognosis has not yet been clarified.

The aim of this study was to assess the superiority of 3D flexible thoracoscope over 2D thoracoscope in prone‐position TE to investigate whether 3D thoracoscope can improve short‐ and long‐term surgical outcomes for patients with esophageal cancer. This study will provide important insights into the potential benefits of 3D endoscopes in the surgical treatment of esophageal cancer, which could have a significant impact on patient care and improve overall prognosis.

## 2 MATERIALS AND METHODS

### 2.1 Approval for human experiments

Written informed consent was obtained for the surgical procedures prior to the thoracoscopic esophagectomy. All methods of this study were carried out in accordance with relevant guidelines and regulations. The present study protocol was approved by the Ethics Committee of Tokai University School of Medicine (Approval No. 18R236) and performed with an opt‐out option, as explained in instructions posted on the website of the hospital.

### 2.2 Patients

**FIGURE 1:**
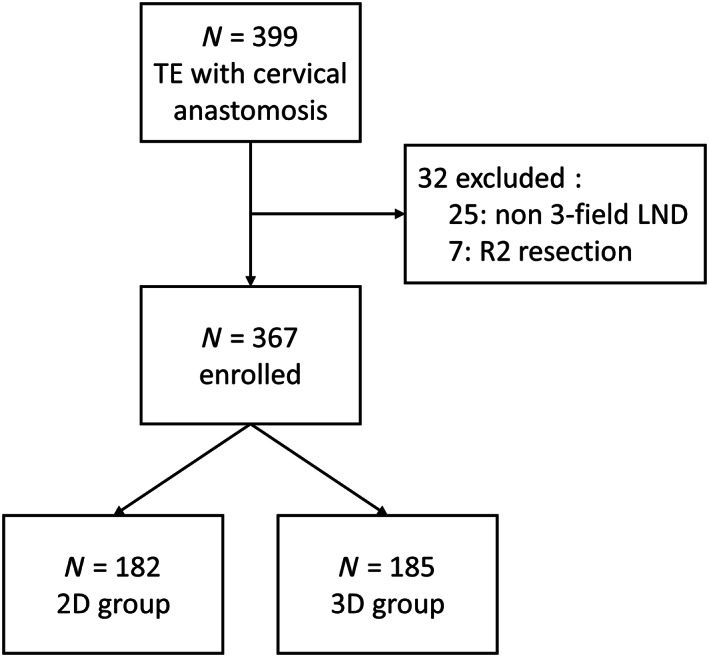
Patient flow of the study. Of the 399 patients who received TE, 32 were excluded and 367 were enrolled; they were divided into a 2D group of 182 patients and a 3D group of 185 patients. LND lymph nodes dissection; TE, thoracoscopic esophagectomy.

Figure 1 shows the patients flow. 399 patients who underwent TE with cervical anastomosis for esophageal cancer at Tokai University Hospital between January 2009 and October 2018 were collected. Patients who did not undergo three‐field LND and patients who were found to have gross intraoperative remnants were excluded. Finally, a total of 367 patients were enrolled: 182 patients underwent with 2D thoracoscopy (2D group), and 185 patients with 3D thoracoscopy (3D group). Preoperative computed tomography and upper gastrointestinal endoscopy with biopsy were performed for all patients to determine the tumor staging. Patients with a preoperative diagnosis of clinical T2 and deeper or positive LN metastasis status underwent preoperative chemotherapy with 5‐fluorouracil and cisplatin (CF) or docetaxel, 5‐fluorouracil, and cisplatin (DCF).

### 2.3 Mediastinal lymph nodes

Esophageal cancer was staged according to TNM classification by the Union for International Cancer Control (UICC) 8th edition.^16^ The Japanese classification of esophageal cancer, 11th edition,^17^ was used for analyzing the LN station. In addition, we classified the retrieved LNs into five zones of origin: cervical, upper mediastinal, middle mediastinal, lower mediastinal, and abdominal.^18^ The middle mediastinal zone and the lower mediastinal zone were divided by the caudal margin of the inferior pulmonary vein. Supraclavicular LN metastasis was not considered as distant metastasis and still judged to be resectable according to the present Japanese esophageal cancer treatment guideline.^19^

### 2.4 Surgical procedures

All the patients enrolled underwent TE in a prone‐position, and the surgery was initiated using thoracoscopy. Briefly, patients were placed under general anesthesia in a semi‐supine position with the right arm raised, followed by full prone position with bed rotation. The patients underwent esophageal mobilization and mediastinal LN dissection under thoracoscopy.^20^ After cervical LND, including bilateral supraclavicular LND, an anastomosis was performed on the left side of the neck, typically using a gastric conduit via retrosternal route. 3D thoracoscope was introduced at our institution in 2015, since then, all patients undergone thoracoscopic surgery using the 3D thoracoscope. Both 2D and 3D scopes were flexible types and there was no difference in resolution. Other than the change from 2D to 3D thoracoscope, all surgical procedures in the study period was performed by the same surgical team and also pre/postoperative management remained the same throughout the study period.

### 2.5 Statistical analysis

Surgical outcomes, the numbers of retrieved LNs, and patient outcomes were compared between the 3D and 2D groups. In particular, the presence of regional LN recurrence was investigated. The numbers of retrieved LNs and LN recurrences were examined separately for each zone.

Correlations with categorizable variables were evaluated using a chi‐square test and the Fisher exact test, while those with continuous variables were evaluated using the Mann–Whitney *U* test. The Student's *t* test or Welch *t* test was used to compare the numbers of dissected LNs after checking for equal variances using the Levene test. To assess factors capable of predicting regional LN metastasis, a multivariable logistic regression analysis was performed after checking the multicollinearity of each factor. The survival times were assessed using cox regression analysis. The statistical examinations were performed using SPSS 26.0 (IBM SPSS, New York). All tests were two‐sided, and *p* values <.05 were considered to indicate statistical significance.

## 3 RESULTS

### 3.1 Short‐term surgical outcomes

The clinical and oncological characteristics for each group are shown in Table 1. Sex, tumor location, and histology did not differ significantly between the 3D group and the 2D group except for age was significantly higher in the 3D group (*p* = .008). Although clinical T category and Stage did not differ between the groups, clinical N category was significantly advanced in the 3D group compared to 2D group (*p* = .03). As a result, preoperative treatment was also significantly more frequent in the 3D group than the 2D group (*p* = .001).

**TABLE 1:** Clinical and oncological characteristics of the patients.

	2D (*N* = 182)	3D (*N* = 185)	*p*‐value
Age (year, median) (range)	66 (43–84)	69 (26–82)	.008^a^
Sex (male, %)	154 (84.6)	159 (85.9)	.41^b^
Histology			
SCC/adenoca.	173/9	173/12	.34^b^
Tumor location			
Upper/middle/lower/EGJ	20/101/54/7	26/91/64/4	.40^b^
cT^c^			
1, 2/3, 4a	119/63	108/77	.10^b^
cN^c^			
0, 1/2, 3	158/24	146/39	.03^b^
cStage^c^ (%)			
I, II/III, IVA, IVB	121/61	110/75	.1^b^
Neoadjuvant therapy			
None/CF/DCF/CRT	121/48/9/4	97/46/31/11	.001^b^

Abbreviations: CF, cisplatin and 5‐fluorouracil; CRT, chemoradiotherapy; DCF, docetaxel, cisplatin and 5‐fluorouracil; EGJ, esophagogastric junction; SCC, squamous cell carcinoma.

^a^ Mann–Whitney *U* test.

^b^ A chi‐square test.

^c^ UICC 8th.

Surgical outcomes for the patients in both groups are shown in Table 2. The thoracoscopic operation time was significantly shorter and blood loss was significantly lesser in the 3D group compared to 2D group (*p* < .001). The incidence of postoperative complications did not differ significantly, nor did the incidences of recurrent laryngeal nerve paralysis, chylothorax, or pneumonia. The R1 resection rate was significantly higher in the 2D group (*p* = .003). No significant difference in postoperative hospital stay was observed between the groups.

**TABLE 2:** Comparison of surgical outcomes.

	2D (*N* = 182)	3D (*N* = 185)	*p‐*value
Ope. time (min, median) (range)	222 (120–390)	208 (106–392)	.02^a^
Blood loss (mL, median) (range)	23 (1–332)	15 (2–288)	<.001^a^
Postope. complications (%)			
Any	107 (58.8)	108 (58.3)	.51^b^
RLNP	53 (29.1)	40 (21.6)	.06^b^
Chylothorax	8 (4.4)	2 (1.1)	.05^b^
Pneumonia	31 (17.0)	34 (18.4)	.42^b^
R1 (%)	17 (9.3)	4 (2.1)	.003^b^
Hospital stay (day, median) (range)	26 (14–177)	30 (2–284)	.29^b^

Abbreviation: RLNP, recurrent laryngeal nerve paralysis.

^a^ Mann–Whitney *U* test.

^b^ A chi‐square test or the Fisher exact test.

### 3.2 LND and recurrence

**FIGURE 2:**
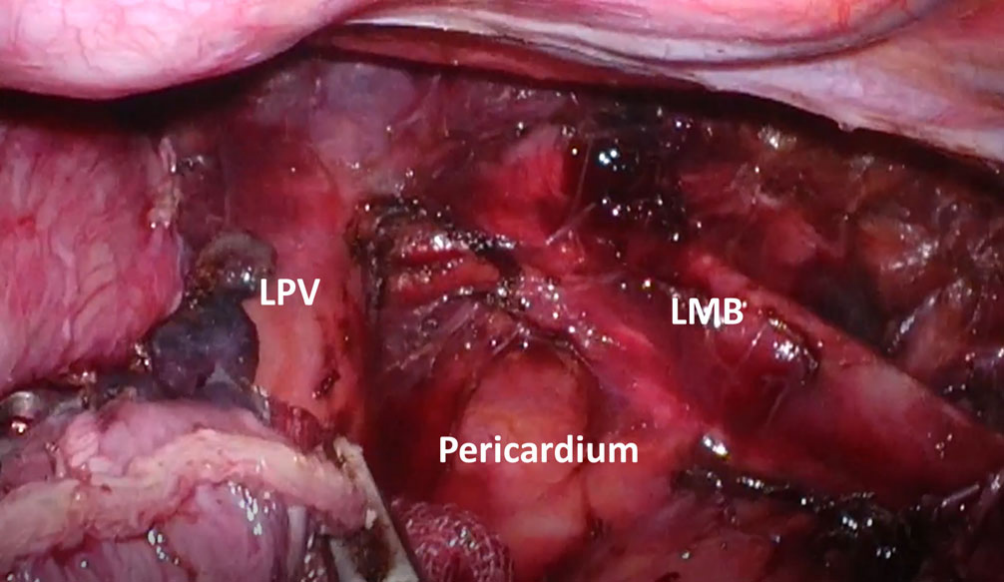
Dissection of LN station 109L. The operative field after dissection of the lymph node at the inferior margin of the LMB (109L) is shown. We believe that depth perception by the 3D scope can play an important role in the mediastinal dissection in this area. LMB, left main bronchus; LPV, lower pulmonary vein.

Table 3 shows the numbers of retrieved LNs in both groups according to zone or station. The total numbers of retrieved mediastinal LNs were significantly higher in the 3D group (*p* < .001), as well as the numbers of retrieved LNs in the upper and lower mediastinal zones was significantly higher in the 3D group (*p* < .001 and *p* = .004, respectively). The numbers of retrieved LNs from the thoracic paratracheal LN (106recL, 106recR, and 106tbL), left main bronchus LN (No. 109L) (Figure 2) and posterior mediastinal LN (No. 112) stations were significantly higher in the 3D group than the 2D group (*p* < .001, *p* = .004, and *p* = .008, respectively). On the other hand, the numbers of retrieved LNs from the cervical paraesophageal LN (No. 101L/R) station was significantly lower in the 3D group than the 2D group (*p* = .001).

**TABLE 3:** The number of retrieved LNs according to LN zone and station.

	2D (*N* = 182)	3D (*N* = 185)	*p*‐value
Chest	23.1	27.4	<.001
Mediastinal LN zone			
Upper	9.3	12.7	<.001
Middle	10.1	10.3	.71
Lower	2.6	3.5	.004
LN station			
No. 101	3.2	2.2	.001
106recL	2.6	4.2	<.001
106recR	2.3	3.2	<.001
106tbL	2.2	3.1	.001
No. 109L	2.0	2.6	.004
No. 112	1.1	1.6	.008

*Note*: All values are means, and all *p*‐values were calculated with the Student's *t* test or Welch *t* test after checking for equal variances using the Levene test.

Abbreviation: LN, lymph node.

Table 4 shows the recurrences in both groups, especially regional LN recurrences, according to the LN regions, zones, or stations. The incidence of overall recurrence and regional LN recurrence was lower in the 3D group compared with the 2D group (*p* = .004 and *p* = .01, respectively). The incidence of mediastinal LN recurrence was also significantly lower in the 3D group than the 2D group (*p* = .005). The rate of cervical LN recurrence was not significantly different between the groups. The numbers of recurrences in the middle mediastinal zone and at the No. 109L station were significantly lower in the 3D group compared than the 2D group (*p* = .001 and *p* = .004, respectively).

**TABLE 4:** LN recurrence according to LN region, zone, and station.

	2D (*N* = 182)	3D (*N* = 185)	*p*‐value
Overall recurrence (%)	70 (38.5)	46 (24.9)	.004
LN recurrence	52 (28.6)	33 (17.8)	.01
LN Region			
Mediastinal	36 (19.8)	18 (9.7)	.005
Cervical	18 (9.9)	12 (6.5)	.159
Abdominal	12 (6.6)	6 (3.2)	.106
Mediastinal LN zone			
Upper	19 (10.4)	11 (5.9)	.083
Middle	18 (9.9)	4 (2.2)	.001
Lower	13 (7.1)	6 (3.2)	.073
LN station			
No. 101	13 (7.1)	6 (3.2)	.073
No. 106	16 (8.8)	11 (5.9)	.2
No. 109L	16 (8.8)	4 (2.2)	.004
No. 112	12 (6.6)	5 (2.7)	.063

*Note*: All *p*‐values were calculated using a chi‐square test or the Fisher exact test.

Abbreviation: LN, lymph node.

### 3.3 Prediction of lymph node recurrence and survival

A multiple logistic regression analysis was performed to assess the risk factors for mediastinal LN recurrence after esophagectomy. The multivariate analysis included 3D or 2D in addition to age, sex, tumor location, histology, clinical T category, clinical N category, neoadjuvant therapy, and postoperative tumor residual, all of which were regarded as risk factors for recurrence. Of these, the logistic regression analysis determined that the use of a 2D scope was a significant independent factor for LN recurrence in the middle mediastinal zone in addition to neoadjuvant therapy and postoperative tumor residual (Table 5). In addition, the impact of different types of preoperative treatment was examined. The recurrence rates for patients who received CF or DCF as preoperative treatment were 11.7% and 10%, respectively, with no significant difference (*p* = .77) (data not shown in table).

**TABLE 5:** Univariable and multivariable analyses for middle mediastinal LN recurrence.

	Number of patients	Mediastinal LN recurrence	Univariable analysis		Multivariable analysis	
			RR (95% CI)	*p*	RR (95% CI)	*p*
Age						
≤ 68	196	14	1			
> 68	171	8	0.63 (0.26–1.56)	.22		
Sex						
Male	313	19	1			
Female	54	3	1.09 (0.31–3.84)	.59		
Tumor location						
Upper	46	3	1			
Middle	192	12	0.95 (0.25–3.53)	.58		
Lower	118	7	0.90 (0.23–3.65)	.56		
EGJ	11	0	NA	.51		
Histology						
SCC	346	22	1	.26		
Adenoca.	22	0	NA			
cT category						
cT1, 2	227	10	1		1	
cT3, 4a	140	12	2.03 (0.85–4.84)	.08	1.11 (0.37–3.30)	.84
cN category						
cN0, 1	304	17	1			
cN2, 3	63	5	1.45 (0.51–4.10)	.32		
Neoadjuvant therapy						
None	218	7	1		1	
Chemotherapy, CRT	149	15	3.37 (1.34–8.49)	.007	4.71 (1.56–14.2)	.006
Postope. tumor residual						
R0	346	14	1		1	
R1	21	8	14.5 (5.20–40.8)	<.001	16.9 (4.38–66.6)	<.001
Type of thoracoscopy						
3D	185	4	1		1	
2D	182	18	4.97 (1.64–14.9)	.001	4.15 (1.26–13.7)	.01

*Note*: Univariable analysis was performed with a chi‐square test or the Fisher exact test, and multivariable analysis was performed with multivariable logistic regression analysis.

Abbreviations: CI, confidence index; CRT chemoradiotherapy; EGJ, esophagogastric junction; LN, lymph node; NA, not assessed; RR risk ratio; SCC, squamous cell carcinoma.

**FIGURE 3:**
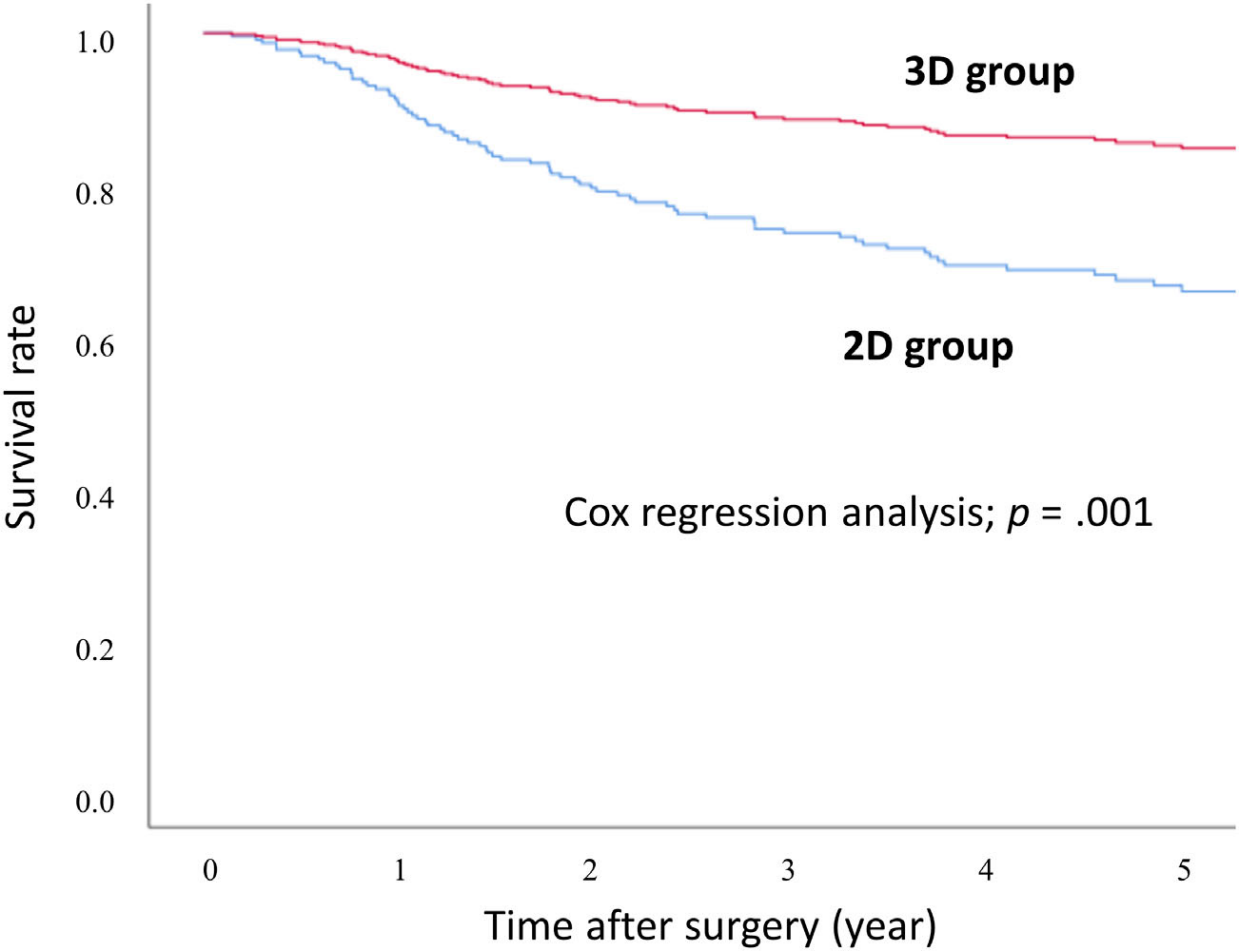
Overall survival for patients operated by 3D scope and 2D scope. Results of cox regression analysis with each factor as a covariate showed significantly better survival in the 3D group compared to the 2D group.

Long‐term prognosis was evaluated in 306 patients after excluding 41 other disease deaths and 21 R1 resections. The use of 3D thoracoscopy, age, cT category, cN category, and presence and type of preoperative treatment were incorporated as covariates and analyzed for survival using cox regression analysis. Significantly better prognosis was observed in the 3D group than in the 2D group (*p* = .001). Results of the survival analysis performed for each group are shown in Figure 3.

## 4 DISCUSSION

In the present study, we demonstrated that utilization of 3D thoracoscope for prone‐position TE could reduce the operative time and blood loss in patients with esophageal cancer. Postoperative complications and postoperative hospital stays were not different between the 3D group and the 2D group. However, 3D thoracoscope may have the potential to improve mediastinal LND accuracy and survival outcomes for patients with esophageal cancer.

First, the safety and ease of the surgical procedure is discussed in terms of operative time, blood loss, and postoperative complications. A simulation‐based study reported that the use of 3D scope provided an advantage on depth perception, compared with 2D scope, enabling good results regarding accuracy and speed of work.^21^, ^22^ For laparoscopic surgery, several studies had compared 3D scopes with 2D scopes, but no clear advantages were pointed out.^23^, ^24^, ^25^ For esophageal cancer surgery, Hou et al. showed that the use of 3D thoracoscope during TE significantly reduced the operative time, with no difference in postoperative complications or postoperative hospital stay, in a prospective study enrolling 154 patients.^13^ Yamashita et al. reported similar results in a retrospective study for 104 patients.^14^ In another retrospective study, Li et al. reported a shorter thoracoscopic surgery time, less thoracoscopic blood loss, and a shorter postoperative hospital stay with the use of 3D compared to 2D endoscope.^15^ In the present study, no reduction in postoperative hospital stay was observed, but reductions in operative time and blood loss were observed in the 3D group, as shown in Table 2.

Next, the accuracy of the surgical technique is discussed in terms of the number of dissected LNs. While two of the three aforementioned previous studies reported no differences in the numbers of dissected LNs,^14^, ^15^ we demonstrated that the numbers of dissected mediastinal LNs were significantly higher in the 3D group than in the 2D group, as shown in Table 3. A detailed analysis of the LN stations showed that the numbers of dissected LNs significantly increased in areas where a field of view was difficult to obtain, such as 109L and 112, or in areas requiring precise manipulation, for example, around the recurrent laryngeal nerve (106recL/recR/tbL). For LN station No. 101, which was dissected from the cervical incision, the number of retrieved LNs was significantly lower in the 3D group. This could be attributable to the fact that LNs at the cervicothoracic border were sufficiently dissected by thoracoscopic manipulation using the 3D thoracoscope. These very interesting results were likely caused by stereovision, which was the great advantage of using 3D endoscopes. Specifically, stereovision enables the surgeon to understand the depth of space available and to perform more reliable operations.

Finally, we discuss the differences in long‐term prognosis resulting from different surgical techniques. The impact of use of 3D thoracoscope during surgery on postoperative recurrence and prognosis for esophageal cancer patients had not yet been reported before. To the best of our knowledge, for the first time our current study showed significantly fewer mediastinal LN recurrences, especially LN recurrences in the middle mediastinal zone with the assistance of 3D thoracoscope for esophageal cancer patients, as shown in Table 4. Multivariate analysis, as shown in Table 5, revealed that the use of a 2D scope was an independent risk factor for middle mediastinal LN recurrence, in addition to cases that received preoperative chemotherapy, which means that they were advanced cases, and incomplete resection. The use of a 3D thoracoscope may facilitate surgeons to perform precise mediastinal LND in stereoscopic visualization. The accuracy of the No. 109L station LND, which are particularly important for understanding the depth of space intraoperatively, may led to the lower recurrence rate that was observed. In this study, while the numbers of middle mediastinal LN dissected were comparable between the groups, the number of middle mediastinal LN recurrences was significantly lower in the 3D group than the 2D group. Although the numbers of LN dissected were important, we consider the possibility that local control was achieved by dissecting the LN without damaging the capsule and preserving the LN structure through 3D stereoscopic visualization. The reduction in regional LN recurrence may also result in better survival outcomes among patients undergoing prone‐position TE using a 3D thoracoscope.

This study had several advantages and limitations. The first of the advantages was that all of the patients underwent TE using the same technique. Second, all surgical procedures in the study period were performed by the same surgical team. We had already established TE prior to the study period, so that there was little bias arising from the technique used for the surgery in the study cohort. Third, almost all patients with Stage II and Stage III cancer had routinely received neoadjuvant chemotherapy, so that there was little preoperative treatment bias. The first limitation was that our study was designed as a retrospective investigation performed at a single center. Second, a bias arising from the difference in time periods may exist. However, we believe that any biases are likely to be minimal because the surgical technique itself had already been established by our team at the beginning of the study and similar procedures were performed during the periods. Although the difference in the percentage of types of preoperative chemotherapy between the 2D and 3D groups cannot be ignored, we confirmed that the rate of middle mediastinal LNs did not change depending on the type of preoperative treatment. To minimize this bias, multivariate analysis was used to analyze long‐term prognosis. Third, we used a flexible scope, but the present results might not be applicable to a rigid scope. Robot‐assisted surgery, which is now widely available, uses a rigid scope. Consequently, the present results might not be applicable to robot‐assisted surgery. However, robot‐assisted surgery also provides a sense of depth and stereoscopic vision, so that, robot‐assisted surgery is expected to be capable of performing precise mediastinal LND, similar to the presently reported results.

The results of this study suggest that the use of 3D thoracoscopes in supine‐position TE may provide better results in clinical practice, and therefore the use of 3D thoracoscopes is recommended, although some problems remain regarding the supply and cost of medical equipment.

In conclusion, prone‐position TE performed using a 3D thoracoscope can improve the accuracy of mediastinal LND and postoperative survival without increasing postoperative complications in esophageal cancer patients. These results have rarely been reported before, and this is the first report of these results, especially regarding long‐term prognosis. Based on the results of our study, we recommend the use of a 3D scope when performing thoracoscopic TE in clinical practice.

## AUTHOR CONTRIBUTIONS

**Kohei Kanamori:** Conceptualization (lead); data curation (lead); formal analysis (lead); investigation (lead); methodology (lead); project administration (equal); writing – original draft (lead); writing – review and editing (equal). **Kazuo Koyanagi:** Conceptualization (lead); funding acquisition (lead); methodology (lead); project administration (lead); supervision (lead); writing – review and editing (equal). **Soji Ozawa:** Data curation (supporting); writing – review and editing (equal). **Junya Oguma:** Data curation (supporting); writing – review and editing (equal). **Akihito Kazuno:** Data curation (supporting); writing – review and editing (equal). **Yamato Ninomiya:** Data curation (supporting); writing – review and editing (equal). **Miho Yamamoto:** Data curation (supporting); writing – review and editing (equal). **Yoshiaki Shoji:** Data curation (supporting); writing – review and editing (equal). **Kentaro Yatabe:** Data curation (supporting); writing – review and editing (equal). **Masaki Mori:** Conceptualization (supporting); validation (supporting); writing – review and editing (equal).

## FUNDING INFORMATION

No funding.

## CONFLICT OF INTEREST STATEMENT

The authors declare no conflict of interest.

## ETHICS STATEMENT

The protocol for this research project has been approved by a suitably constituted Ethics Committee of the institution and it conforms to the provisions of the Declaration of Helsinki. Ethics Committee of Tokai University School of Medicine, Approval No. 18R236. It is performed with an opt‐out option, as explained in instructions posted on the website of the hospital. The manuscript has not been submitted to more than one journal for simultaneous consideration.

## Data Availability

The data set used to conduct this research will be made available on request.
